# Comment on "Evaluating the reliability of YouTube videos as an educational source on heart failure"

**DOI:** 10.1590/1806-9282.20260493

**Published:** 2026-08-24

**Authors:** Huseyin Durmaz, Semiha Durmaz

**Affiliations:** 1 Konya City Hospital, Department of Cardiovascular Surgery – Konya, Türkiye.; 2 Konya City Hospital, Department of Internal Medicine – Konya, Türkiye.

Dear Editor,

I have read with interest the article entitled "Evaluating the reliability of YouTube videos as an educational source on heart failure"^1^ recently published in the journal Revista da Associação Médica Brasileira by Dr. Bulguroglu and Dr. Erdogan. The study is an important and timely research in that it evaluates the quality levels of videos related to heart failure and emphasizes their usability for informing patients.

The data presented in the study show that some scales are used in the evaluation of the quality and reliability of videos related to heart failure. In this sense, it is seen that valuable scales such as the JAMA (Journal of the American Medical Association), the DISCERN (Quality Criteria for Consumer Health Information), and the Global Quality Scale (GQS) are used in the current study. However, I think there are some points that need to be addressed in this study.

There are different measurement tools that can be used to evaluate the quality and reliability of videos. One of them is the Video Information and Quality Index (VIQI). VIQI consists of four dimensions: information flow (VIQI-1), information accuracy (VIQI-2), quality [including components such as visual material, animation, interview, video title, and summary] (VIQI-3), and sensitivity [level of consistency between video title and content] (VIQI-4)^2^. Each parameter is scored between 1 and 5, with higher scores indicating better video quality. Therefore, I believe that including the VIQI scale in the study will contribute to a more comprehensive and multidimensional evaluation of the quality and reliability levels of videos.

Research on social media and health communication reveals that the production and presentation methods of online health content can be influenced by cultural, technological, and regional dynamics^3^. In this context, addressing these parameters in the study may contribute to a more contextually sound foundation of the findings and to increasing the overall comprehensibility of the article.

In conclusion, the study by Dr. Bulguroglu and Dr. Erdogan presents valuable and noteworthy findings in terms of evaluating the quality levels of videos related to heart failure. I believe that further research in this area will make significant contributions to the literature, and I hope that the authors will take these suggestions into account in their future work. Thank you for giving me the opportunity to share my thoughts on this important study.

Yours truly

## Data Availability

The datasets generated and/or analyzed during the current study are available from the corresponding author upon reasonable request.
